# A Rare Case of Thiamine Deficiency Leading to Dry Beriberi, Peripheral Neuropathy, and Torsades De Pointes

**DOI:** 10.7759/cureus.48853

**Published:** 2023-11-15

**Authors:** Trevor Phinney, Kathlyn Callenius, Madhurmeet Singh, Kristin Juhasz, Mirsha Stiven, Jeffrey Tseng

**Affiliations:** 1 Neurology, University of Pittsburgh Medical Center Hamot, Erie, USA; 2 Neurology, University of Pittsburgh Medical Center Hamot, Erie , USA; 3 Cardiology, University of Pittsburgh Medical Center Hamot, Erie, USA; 4 Emergency Medicine, University of Pittsburgh Medical Center Hamot, Erie, USA

**Keywords:** thiamine deficiency, wet beriberi, dry beriberi, torsades de pointes, prolonged qtc

## Abstract

Thiamine (vitamin B1) is an essential nutrient and one of the eight B vitamins. As a water-soluble vitamin, thiamine is not stored; therefore, a balanced diet is required to ensure adequate intake of this essential vitamin. Thiamine deficiency is known to cause both wet and dry beriberi, but rarely in combination. Thiamine deficiency has also been known to cause QTc prolongation, but the mechanism remains unclear. In the most severe cases, this can lead to the lethal arrhythmia of torsades de pointes. This case describes a patient who became malnourished after a closed head injury and initially presented with seizure-like activity and syncopal episodes with nonspecific numbness. He was found to have prolonged QTc, leading to torsades de pointes requiring an implanted cardioverter defibrillator. With extensive workup, including genetic testing, the patient was found to have indetectable thiamine levels. With supplementation, the patient no longer had any recorded ventricular arrhythmias, and neurological function improved with only residual tingling in the hands. This case emphasizes the profound effects of thiamine deficiency and why this should be included in our differential diagnosis for patients presenting with the sequelae of the signs and symptoms discussed.

## Introduction

Thiamine (vitamin B1) deficiency, also called beriberi, classically has two presentations: wet and dry. Wet beriberi typically presents with heart failure and cardiomegaly, while dry beriberi presents with a peripheral neuropathy that often mimics Guillain-Barré syndrome. Although these presentations are the most common, thiamine deficiency can also lead to other cardiac arrhythmias and Wernicke-Korsakoff syndrome [1, 2]. Typically, the cardiac conduction issues with thiamine-deficient patients are mild and do not lead to any further investigation or intervention. Nonetheless, among the cardiac arrhythmias seen in thiamine deficiency is prolonged QT syndrome [3, 4]. We present a unique case of a patient with both cardiac and neurological symptoms who developed torsades de pointes requiring defibrillator implantation and a severe sensorimotor polyneuropathy mimicking Guillain-Barré syndrome. Few studies report patients presenting with symptoms of both wet beriberi and dry beriberi. In the available reports, symptoms involved right-sided heart failure and axonal polyneuropathy [5, 6]. This case was previously presented at the annual University of Pittsburgh Medical Center Hamot Research Conference on April 29, 2022.

## Case presentation

A 34-year-old male presented to the emergency department (ED) with seizure-like events and syncopal episodes for nine months and non-specific numbness and tingling for two years. He was admitted for further evaluation. Three years before admission, he suffered a fall from a roof, which resulted in a closed head injury. Since that event, the patient felt that his health had deteriorated. He became increasingly malnourished, most likely due to his diet. During this period, he ate an average of six to seven meals per week and drank at least six beers per week, approximately one beer daily. He incurred a 30-pound weight loss over the past three years. Along with his lack of oral intake, he had excessive nausea and vomiting for six months, which was being managed as an outpatient by gastroenterology.

**Figure 1:**
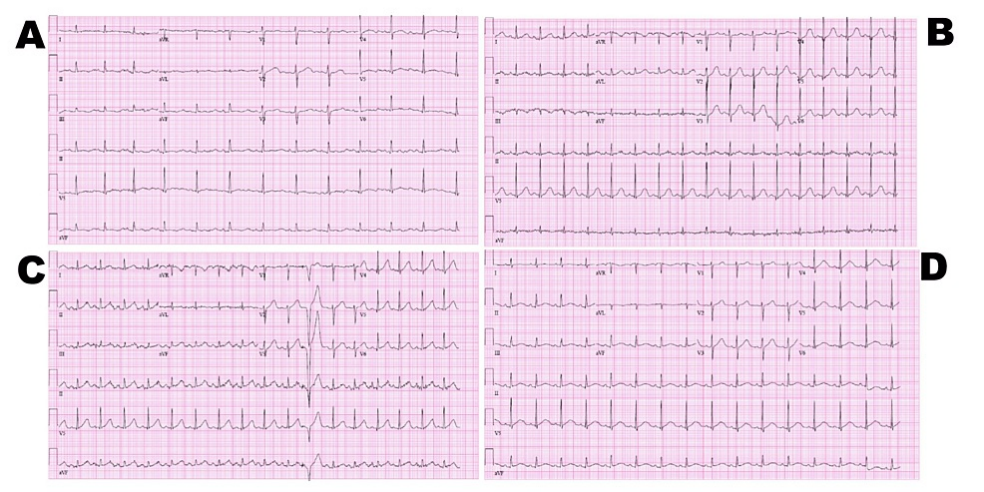
Electrocardiograms (EKGs) obtained during hospitalization A) Initial EKG demonstrated a significantly prolonged QTc of 625 milliseconds (ms). B) Continued prolonged QTc of 523 ms 17 days later. C) After placement of an implanted cardioverter defibrillator (ICD), QTc remained prolonged at 496 ms (day 23). D) Prior to discharge, the patient had a prolonged QTc of 530 ms (day 25).

During his initial admission for these seizure-like episodes, studies revealed a normal brain and cervical spine MRI, as well as a normal electroencephalogram (EEG). Only subjective sensory findings were noted on the physical exam. He was discharged home with outpatient follow-up. He returned two weeks later for intractable nausea. His nausea and vomiting improved with intravenous fluids as well as antiemetic medication. He received potassium replacement as well. An electrocardiogram (EKG) done at that time was notable for a prolonged QTc (QT interval corrected for heart rate) of 580 milliseconds (ms). No prior EKGs were available for comparison. His home prescription for ondansetron was discontinued in the setting of prolonged QT, but no other treatment for his prolonged QT was administered at that time. Esophagogastroduodenoscopy (EGD) showed duodenal erosions; the patient was discharged two days later with anti-nausea medication (metoclopramide), proton pump inhibitor (pantoprazole), folic acid, and pain control (ibuprofen), though ibuprofen is generally not recommended with EGD findings. One week later, the patient had further syncopal episodes with generalized shaking. Continuous EEG monitoring was performed to capture one of his seizure-like episodes. The EEG was normal. However, on this admission, his rhythm strip in the ED showed a very prolonged QTc of 625 ms (Figure 1A), sinus tachycardia, and frequent premature ventricular contractions (PVCs).

Shortly after admission, he was noted to have an episode of PVC-induced (“R on T”) torsades de pointes. Of note, the patient was also being treated with IV metoprolol for his sinus tachycardia. Cardiology was consulted, and EKGs were repeated, which continued to demonstrate profound QTc prolongation. An echocardiogram revealed a structurally normal heart. Potassium and magnesium were slightly low at 3.2 and 1.3, respectively. In the absence of any obvious reversible QTc-prolonging abnormalities (severe electrolyte derangement, intra-cranial pathology, cardiomyopathy/congestive heart failure, medication use, etc.), a secondary prevention implanted cardioverter defibrillator (ICD) was recommended. Repeated EKGs continued to show prolonged QTcs of 622 ms, 523 ms (Figure 1B), and 496 ms (Figure 1C), despite normalization of serum potassium and magnesium. Of note, the patient had no known family history of ventricular arrhythmias, sudden cardiac death, or ICD or pacemaker implantation. A dual-chamber ICD was implanted five days after admission, and the patient was started on nadolol for suspected occult long QT syndrome with plans for outpatient genetic testing. An EKG performed before discharge demonstrated a continued prolonged EKG of 530 ms (Figure 1D). He was discharged to a rehabilitation facility.

**Figure 2:**
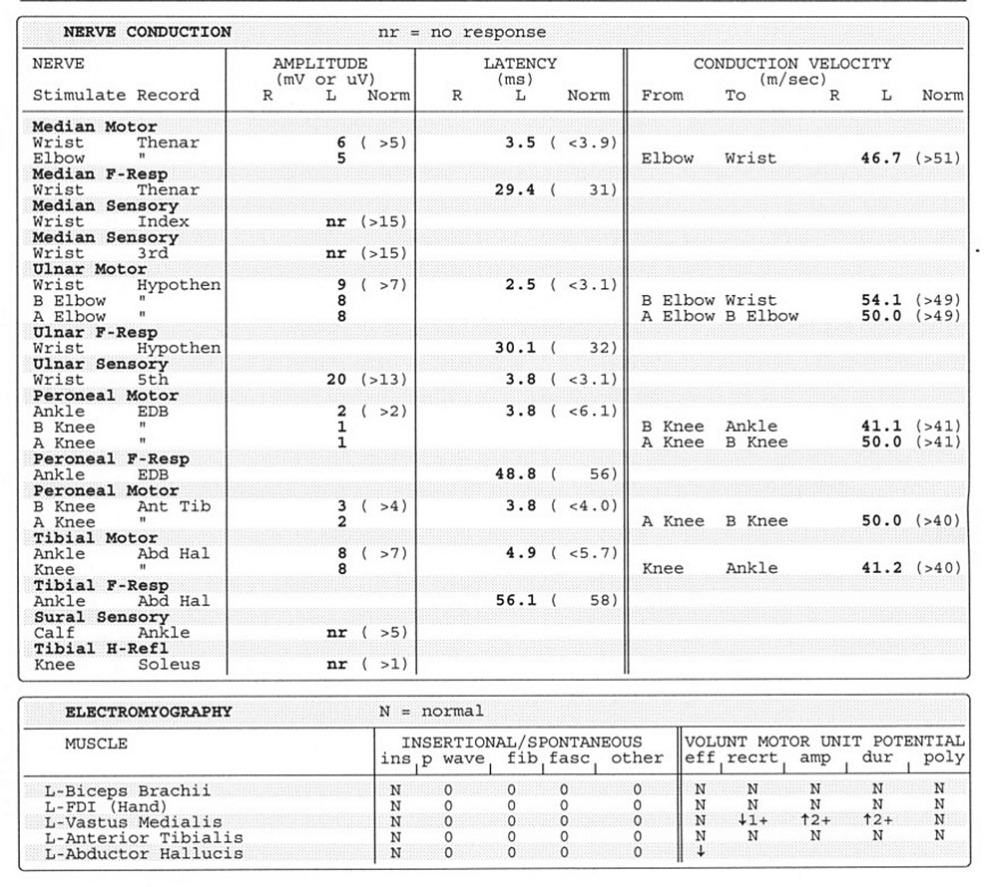
Electromyography report Summary: All sensory nerve action potentials (SNAPS) were unelicitable or had prolonged distal latencies. The peroneal compound muscle action potential (cMAP) amplitude was low. Needle electrode examination revealed an increased proportion of high amplitude, long duration, and fast-firing motor unit potentials (MUPs) in the left vastus medialis muscle. Interpretation: There is electrodiagnostic evidence for the following: Severe sensory >>>>motor peripheral polyneuropathy consistent with the diagnosis of thiamine deficiency; mild to moderate remote left L3/4 radiculopathies; there is no electrodiagnostic evidence for Guillain-Barre syndrome (acute inflammatory demyelinating polyradiculoneuropathy (AIDP)).

He returned to the ED one week later with sudden gait ataxia and a self-reported worsening of numbness and tingling. He reported that the sensory changes had been present for approximately two years; however, the gait ataxia was new. A neurologic exam now revealed areflexia and bilateral sensory gradients, reminiscent of a clinical Guillain-Barré-like syndrome. Multiple labs were ordered at this time to assess the changes in the neurologic exam. Given his history of poor diet, he had multiple vitamin levels drawn, including vitamin B12, folate, and a thiamine level. His thiamine level was found to be undetectable. Electromyography (EMG) (Figure 2) and nerve conduction studies (NCS) were done, which showed a severe sensory deficit greater than motor peripheral polyneuropathy, consistent with the diagnosis of thiamine deficiency.

No electrodiagnostic evidence for Guillain-Barré-like syndrome was noted on the EMG. All genetic testing for prolonged QT was negative. The patient was initially started on IV thiamine, which caused considerable pain in his IV site; he was then switched to oral replacement. He was discharged back to his rehabilitation facility on oral thiamine supplementation with repeat thiamine levels for one month from the date of discharge, as well as an EKG after thiamine levels were redrawn. He was in rehabilitation for a few weeks before being discharged home.

The patient has been followed up in the clinic since discharge. He has become more active and walks on the treadmill daily. His repeat thiamine level was 246 nmol/L (normal reference range: 78-185 nmol/L). The patient did not follow through with the repeat EKG that was ordered. His defibrillator was interrogated and did not show any further episodes of ventricular tachycardia (VT) or ventricular fibrillation (VF). The patient also did not have any events of tachycardia, with an average heart rate between 80 and 100 beats per minute. He continues to have some tingling in his hands, but he is currently taking gabapentin to help control these symptoms. The patient has seen cardiology in the outpatient clinic and remains on nadolol without side effects.

## Discussion

The patient depicted in this case report presented with convulsive syncopal episodes, prolonged QTc, torsades de pointes requiring secondary prevention defibrillator placement, and acute onset of gait ataxia with worsening sensory symptoms, all likely caused by thiamine deficiency.

This case report illustrates interesting points regarding the need for more in-depth diagnostics for not only neurologic dysfunction but also cardiac conduction issues. As noted above, none of the usual suspects contributing to QTc prolongation were evident in this case, including severe electrolyte abnormalities, medication use, drug abuse, cardiomyopathy, intracranial pathology, hypothermia, etc. Thiamine deficiency leading to significant QTc prolongation has been reported in the past, but the exact mechanism remains unclear. In addition, sinus tachycardia is described as having severe thiamine deficiency, and this patient’s persistent sinus tachycardia at the time of presentation was likely a consequence of this.

The cardiac nature of thiamine deficiency can be traced back years to 1929 when Christiaan Eijkman received the Nobel Prize for his discovery of “antineuritic vitamins” related to chickens and the specific rice they were eating. He was one of many who started the initial investigation of vitamins related to beriberi and how to treat these symptoms [7]. Since that time, many different aspects of cardiomyopathy, arrhythmias, and EKG changes have been observed. However, these cardiac defects are often associated with wet beriberi and have rarely been seen in conjunction with dry beriberi. Although a few studies have studied a combination of wet and dry beriberi, symptoms usually involve right-sided heart failure and axonal polyneuropathy [5, 6]. This patient had such a severe thiamine deficiency that his QTc prolongation led to a potentially lethal arrhythmia of the torsades de Pointes, which required defibrillator placement. This type of severity of EKG change and intervention is extremely uncommon with thiamine deficiency. Neurologically, the patient initially presented with seizure-like episodes, which it turns out were related to his above-noted cardiac arrhythmias, yet it took significant investigation and prolonged EEG monitoring to establish this. When he secondarily presented with sudden gait ataxia, a Guillain-Barré-like syndrome was considered, and our diagnostic approach broadened. These neuropathic complaints are those typical for dry beriberi, with the exception that the patient reported the sensory symptoms had been mild for two years prior to the subacute increase in numbness and ataxia. All central nervous system (CNS) imaging of the brain, cervical, and lumbar spines was benign without any acute or chronic pathology. Lumbar puncture was not performed in this patient as the clinical and laboratory findings led to the diagnosis of thiamine deficiency.

## Conclusions

Thiamine deficiency can present as wet or dry beriberi. Rarely is there such a combination of significant cardiac complications as well as neurologic complications that can all be traced back to severe malnourishment and the resultant severe thiamine deficiency. This case report illustrates the need for more in-depth diagnostics for not only neurologic dysfunction but also cardiac conduction issues. While the recognition of thiamine deficiency causing dry beriberi seems to be increasing due to expanded knowledge on the topic, this case may lead to further investigations related to its prevalence in otherwise unexplained cardiac conditions.
